# Assessing cognitive flexibility in humans and rhesus macaques with visual motion and neutral distractors

**DOI:** 10.3389/fpsyg.2022.1047292

**Published:** 2022-12-20

**Authors:** Pinar Yurt, Antonino Calapai, Roger Mundry, Stefan Treue

**Affiliations:** ^1^Cognitive Neuroscience Laboratory, German Primate Center, Goettingen, Germany; ^2^Georg-August University School of Science, Goettingen, Germany; ^3^LeibnizScienceCampus Primate Cognition, Goettingen, Germany; ^4^Cognitive Ethology Laboratory, German Primate Center, Leibniz Institute for Primate Research, Goettingen, Germany; ^5^Department for Primate Cognition, Georg-August University, Goettingen, Germany

**Keywords:** executive functions, reversal learning, set-shifting ability, visual motion, macaque, foraging, cognitive flexibility

## Abstract

**Introduction:**

Cognitive flexibility is the ability of an individual to make behavioral adjustments in response to internal and/or external changes. While it has been reported in a wide variety of species, established paradigms to assess cognitive flexibility vary between humans and non-human animals, making systematic comparisons difficult to interpret.

**Methods:**

We developed a computer-based paradigm to assess cognitive flexibility in humans and non-human primates. Our paradigm (1) uses a classical reversal learning structure in combination with a set-shifting approach (4 stimuli and 3 rules) to assess flexibility at various levels; (2) it employs the use of motion as one of three possible contextual rules; (3) it comprises elements that allow a foraging-like and random interaction, i.e., instances where the animals operate the task without following a strategy, to potentially minimize frustration in favor of a more positive engagement.

**Results and Discussion:**

We show that motion can be used as a feature dimension (in addition to commonly used shape and color) to assess cognitive flexibility. Due to the way motion is processed in the primate brain, we argue that this dimension is an ideal candidate in situations where a non-binary rule set is needed and where participants might not be able to fully grasp other visual information of the stimulus (e.g., quantity in Wisconsin Card Sorting Test). All participants in our experiment flexibly shifted to and from motion-based rules as well as color- and shape-based rules, but did so with different proficiencies. Overall, we believe that with such approach it is possible to better characterize the evolution of cognitive flexibility in primates, as well as to develop more efficient tools to diagnose and treat various executive function deficits.

## Introduction

In order to survive and reproduce, animals need to be able to flexibly adjust their behavior in response to changes in the environment. The ability to perform these adjustments is known as cognitive flexibility (Highgate and Schenk, 2021) and it is considered to be one of the fundamental components of executive functions (Nyhus and Barceló, 2009; Stoet and Snyder, 2009; Miyake and Friedman, 2012; Diamond, 2013; Manrique and Call, 2015; Glisky et al., 2021). Whether behavioral adjustments are required because an established response has stopped producing the desired outcome (e.g., a monkey that finds no more seeds while browsing through the fallen leaves), or because internal needs have changed (e.g., a monkey feels thirst while foraging), most organisms need to be able to inhibit current behavioral responses (e.g., stop browsing for seeds) so that novel and more appropriate responses can be put in place (e.g., search for a new patch of leaves with potentially more seeds or go to the nearby pond to drink). Cognitive flexibility has therefore been reported in a large variety of species, such as fish (Miletto Petrazzini et al., 2017; Buechel et al., 2018; Fuss and Witte, 2019), birds (Rayburn-Reeves et al., 2013; van Horik and Emery, 2018; Reichert et al., 2020; Aljadeff and Lotem, 2021; Loconsole et al., 2021), insects (Strang and Sherry, 2014; Wenig et al., 2021), mice and rats (Bryce and Floresco, 2021; Caglayan et al., 2021; Odland et al., 2021), octopuses (Bublitz et al., 2021), various mesocarnivores (Stanton et al., 2021), non-human primates (Mahut, 1971; Dias et al., 1996a; Izquierdo, 2004; Kuwabara et al., 2014; Shnitko et al., 2017; La Camera et al., 2018; Watzek et al., 2019; Weiss et al., 2019; Grant et al., 2021), and humans (Xue et al., 2013; Lange et al., 2018; Lawrence-Sidebottom et al., 2020; Takeda and Fukuzaki, 2021; Weiss et al., 2021). Most of the paradigms developed to assess cognitive flexibility fall in one of two categories: set-shifting or reversal-learning (for a review see Uddin, 2021). In both, participants are first taught which of multiple visual stimuli lead to a correct response, through a process of trial and error and based on simple binary correct/incorrect feedback. When this rule is acquired, the rule changes. As participants need to disengage from the ongoing behavior and flexibly adapt to the new association, their response and performance after the rule change is quantified to provide a measure of the participants’ flexibility (Rayburn-Reeves et al., 2017; Shnitko et al., 2017). In more detail, in set-shifting paradigms, like the Wisconsin Card Sorting Test (Grant and Berg, 1948; Nelson, 1976; Stuss et al., 2000; Prentice et al., 2008; Nyhus and Barceló, 2009; Lange et al., 2018; D’Alessandro et al., 2020; Uddin, 2021), human participants are asked to sort cards according to one of several contextual rules (e.g., the card’s shape, color, or number of objects) that are changed unpredictably by the experimenter. In reversal learning tasks, participants learn which one of two stimuli is the correct one (discrimination phase) and once their performance is stable, namely when a certain criterion is met, the rule is reversed and the correct stimulus becomes the incorrect one (reversal phase). While reversal learning approaches (with one binary rule) have been used with both humans and non-human animals, most set-shifting paradigms (with multiple rules) are optimized for humans and do not generalize well to other animals (see Uddin, 2021 for a review). Set-shifting tasks for non-human primates require animals to shift only between two rules (namely the shape and the color of a stimulus), as opposed to human versions of the tasks that require participants to shift between three rules (color, shape, and quantity). As a result, to the best of our knowledge, a direct comparison of set-shifting abilities of humans and non-human primates is not available.

To fill this gap, we developed a task that integrates elements from both reversal learning and set-shifting approaches and that can assess cognitive flexibility of both species without species-specific adaptations. Our multidimensional shifting task (MDS) makes use of two main features: (1) it uses motion as a third visual feature dimension, in addition to color and shape; (2) it employs an array of four stimuli (1 target, 1 distractor and 2 neutral stimuli). With this task, we were able to assess and rank the cognitive flexibility of 11 captive Rhesus macaques and 25 human participants. Our results show that monkeys maintain a high level of engagement with the task within and between sessions. We also found that efficiency in learning the rule is similar across the three features (shape, color, and motion), which suggests that motion is suitable as an additional feature dimension for cognitive assessment of both humans and monkeys. Ultimately, we computed a custom-made index of cognitive flexibility across different types of shifts (intra-dimensional or extra-dimensional); across feature dimensions (shape, color, and motion), and across species (humans and monkeys). We found that humans and monkeys cluster on opposite sides of the scale; that cognitive flexibility is independent from the visual feature used to assess it; and that only humans show intra- to extra-dimensional differences.

## Materials and methods

### Participants

A total of 25 human participants (13 males and 12 females, mean age = 28 years old) and 14 rhesus macaques (*Macaca mulatta*, all male, mean age = 11 years old) were tested. All participants first went through a training procedure and once they completed training, they went forward with the testing. While both training and testing happened on the same day/session for humans, it took on average 6 days for monkeys to complete both training and testing procedures (Supplementary Table S2). The testing part ended when three cycles (discrimination-reversal pairs) from each feature dimension were completed.

Human participants were given written instructions before the experiment and they all read and agreed to a consent form while the participation was completely voluntary and they were informed that they can stop anytime they want. The participants were tested in a light controlled room and they stabilized their head on a chin rest to ensure the same distance from the screen across participants, and they were asked to respond to the task with a mouse click. After the experiment, participants received a compensation that was depending on the length of the session, as well as a performance-based bonus.

We have included 11 monkeys in the analysis because two animals did not interact with the device enough to allow meaningful quantification of performance and technical problems prevented data collection from one other animal. The animals were rewarded with their favorite fluid reward for each correct answer and they received free water, fresh fruits, vegetables, and nuts after each session.

Research with non-human primates represents a small but indispensable component of neuroscience research. The scientists in this study are aware and are committed to the great responsibility they have in ensuring the best possible science with the least possible harm to the animals (Roelfsema and Treue, 2014; Treue and Lemon, 2022). All animal interactions carried out in this study, housing conditions, and the animal care comply with the regulations of the regional government office Niedersaechsisches Landesamt für Verbraucherschutz und Lebensmittelsicherheit (LAVES) under the permit number 33.19–42,502–04-18/2823. The animals were group-housed (two or three animals) in the facilities of the German Primate Center (DPZ) in Goettingen, Germany. The facility provides the animals with an enriched environment including a multitude of toys and wooden structures, natural as well as artificial light and exceeding the size requirements of the European regulations, including access to outdoor space. The animals’ psychological and veterinary welfare was monitored by the DPZ’s staff veterinarians, the animal facility staff, and the lab’s scientists, all specialized on working with non-human primates. The animals were always provided *ad libitum* monkey chow which was dispersed around the cage to give them the opportunity to forage. No invasive procedure was necessary during the development, testing, and the general use of the devices and our experimental procedure.

### Testing apparatus for monkeys

**Figure 1:**
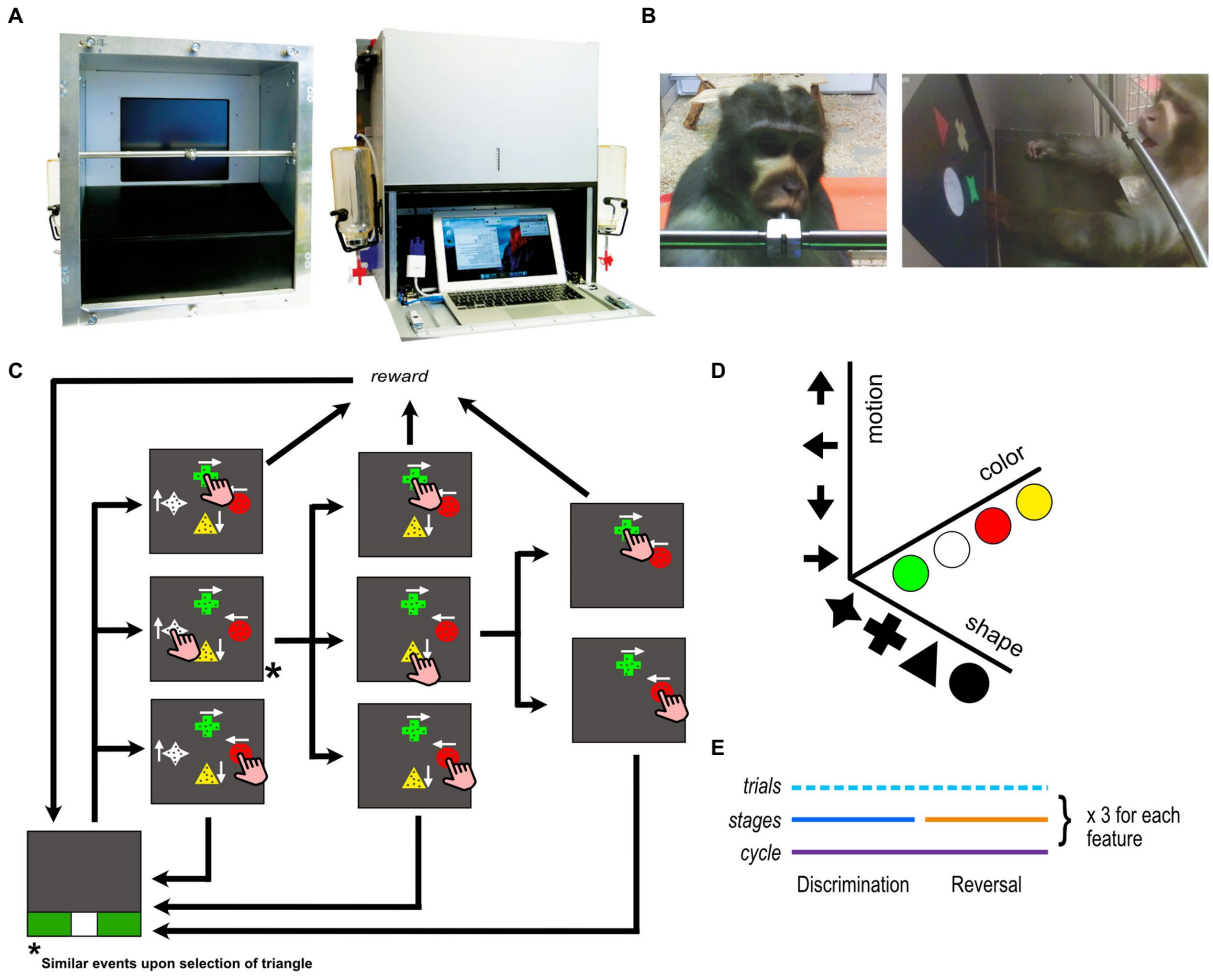
Touchscreen device and experimental task. **(A)** The device seen from the front and the back **(B)** an animal interacting with the device in his home environment seen through the front and side cameras **(C)** the task design. In this particular stage, the attended feature is shape with cross being the target and circle the distractor. If target or distractor is touched, the reward is delivered for target selection only but both options terminate the trial. Different auditory feedback is given for target and distractor touches. On the other hand, if one of the neutral stimuli (star or triangle in this case) is selected, it disappears and the trial continues until either a target or a distractor is chosen as illustrated in the figure. For the sake of simplifying the figure, the sequence of events for selecting a triangle is not illustrated in the figure but the trial would continue in a similar fashion as in the case for the selection of star **(D)** the three feature dimensions used in the experiment **(E)** the timeline of a complete assessment loop.

A custom-made, stand-alone, autonomous, touchscreen device was used for data collection. The device is an updated version of the eXperimental Behavioral Instrument (XBI), that was developed in-house (Calapai et al., 2017; Berger et al., 2018). The XBI can be attached directly to the home enclosure of the animals and can be used to run cognitive experiments, behavioral training, as well as enrichment protocols (Figure 1). The version of the XBI used in this study comprises a centralized computational unit (MacBook Air, Apple – 2018), a 15″-touchscreen, and a custom-made microcontroller to acquire touchscreen information and to control the fluid reward system.

### The experimental task

The experimental task was developed and administered with the open-source software MWorks^1^, which allows for flexible real-time control of a variety of standard and custom-made cognitive tasks. Each trial was initiated by the animal touching the start trial stimulus (white square) located at the bottom of the screen (Figure 1C). Then, four different stimuli were presented on the screen simultaneously, each at a different location, but equally distanced from each other. Each stimulus had one of four different shapes, one of four different colors, and contained a cloud of dots moving in one of four different directions (Figure 1D). In a given trial, each stimulus was composed of a unique combination of these three feature dimensions. One of the stimuli was the *target*, and if touched, the trial terminated, a unique acoustic feedback was provided, and a drop of fluid reward was delivered (fruit juice diluted at 33%, 0.25 mL drop size). Another stimulus was a *distractor* which if touched terminated the trial after delivering a unique acoustic feedback but no fluid reward. The remaining two stimuli were what we call *neutral distractors* that disappeared upon touch without terminating the trial and they did not provide acoustic feedback or fluid reward. The task was composed of discrimination and reversal *stages*, always coupled together (in succession) and comprising together a *cycle* (Figure 1E). At every cycle one of the three features was pseudo-randomly set to be the relevant one for both the discrimination and reversal stages. Once both stages were completed, another cycle was started and another feature was randomly assigned to be the new relevant one. At every trial all four categories of all three features appeared on the screen in randomized combinations, while target and distractor were set to two categories of the relevant feature (for example color yellow and green) until the stage was completed. When a performance of 80% correct responses across the last 10 trials was reached, the discrimination stage was considered to be completed. At this point the second stage of the cycle started, the reversal stage, in which the rewarded and unrewarded categories of the relevant feature were swapped (e.g., circle now represents the target and cross the distractor, see Figure 1C). This rule change occurred without indication to the participants whom can only infer it by the sudden reverse in feedback. After also the reversal was completed, according to the same threshold as the discrimination stage, another cycle began with a different relevant feature (in 80% of the cases) or with the same feature (in 20% of the cases). If the relevant feature in the current cycle was the same as the one in the previous cycle, then we labeled this shift as *intra-dimensional shift*. On the other hand, if the features did not match, we labeled this shift as *extra-dimensional shift*. We have allowed for chance-aided progress in our task such that participants were able to complete a stage without following a strategy, i.e., *via* random interactions. This approach was especially crucial to prevent subjects from being stuck at a particular stage and to potentially decrease the likelihood that animals would disengage from the task when stuck. The stages that are completed by chance were then filtered out before data analyses.

Prior to the testing phase, an automatized training protocol instructed the participants on the basics of the task [AUT from Berger et al. (2018)]. During this training phase participants navigated a series of pre-programmed training steps in which specific elements and rules of the task were gradually introduced based on their performance. They were presented with a more difficult step only when their performance on the current step reached 80% (in the last 10 trials). Specifically, the three visual features employed in our task were added one by one at successive steps, with for instance, stimuli at the first step of the training procedure having the same color and motion but differing only in their shape and then in all three dimensions (shape, color, and motion) at later steps (Shnitko et al., 2017). We used 3 different series of steps (which we refer here as different training types), in which the features were introduced in different orders to account for a potential influence of order on the participant’s performance in the main testing task. AUT1 first trained shape, then color, then motion; AUT2 trained color first, then motion, and finally shape; AUT3 started with motion, then color, and shape at the end.

### Data collection

Human participants were tested in a dedicated psychophysics setup at the Cognitive Neuroscience Laboratory at the German Primate Center, for a single session. They underwent the same procedure as the animals, with the difference that human participants used a mouse instead of a touch screen and the start button in this case was placed in the middle of the screen. Animals were tested opportunistically and on consecutive downtime days from their main experimental routine (for an average of 6 sessions each lasting on average 121 min). During the testing, companion animals were not present or moved to a different section of the enclosure, while testing animals had access to the XBI from the main compartment of their home enclosure. Tested animals were always kept in their home enclosure in visual and acoustic contact with the conspecifics. On average, animals completed the training in the first 3 sessions and the testing continued on following days until data from at least 3 cycles from each feature dimension were collected (Figure 1E). We included in our analysis 11 out of 14 animals (2 animals did not complete the training and 1 animal did not interact with the device) and 25 out of 26 humans (due to technical issues during data collection for 1 participant). All analyses were performed using MATLAB (2020) and R version 4.x, R Core Team (2021) was used for GLM modeling, while the matlab package *gramm* (Morel, 2018) was used for plotting.

### Data analysis

We conducted a series of three statistical analyses. First, we assessed the likelihood of each stage to have been completed by chance (*Chance-level estimation*). We then assessed the number of choices (note that multiple choices can be made in a given trial) needed to complete a given stage, across a number of factors; and evaluated systematic effects of these factors as well as their interactions (*Second-step modeling*). Finally, for each stage that was not solved by chance we measured a simple cognitive flexibility index dividing the total number of stages performed by the total number of trials performed (see Equation 1, *Cognitive flexibility index* – *CFI*). The following sections will describe these three analyses in detail.

### Chance-level estimation

During the experiment, a stage ended when at least eight correct trials out of 10 consecutive trials were performed. At this point the contextual rule was changed, according to mechanics described above (see *Experimental Task*). We evaluated whether stages were completed as a result of a stochastic process or by the participants having reached an understanding regarding the contextual rule. To this end, we fitted a Generalized Linear Model (GLM) with binomial error structure and logit link function (McCullagh and Nelder, 1989) to the sequence of individual touches, with the response being whether the individual touched the target or not, separately for each individual stage. The ultimate aim of this model is to estimate how the probability to touch the target changed over the course of a stage, to estimate the probability to touch the target at the last touch of a stage and whether this probability was significantly above chance (see Supplementary Figure S4, see below for the description). To account for the fact that the probability to touch the target increased within a single trial, we included 1 divided by the number of the available stimuli (logit-transformed; i.e., logit(x) = log(x/(1-x))) as an offset term (McCullagh and Nelder, 1989). To avoid fitting problems with offset terms being infinite, we excluded touches for which the probability of choosing the target was one. We fitted such a model for each individual stage. The reasoning behind this model was the following: if the individual learns over the course of the stage, its probability to touch the target will gradually increase. In this case the slope estimate of the GLM will be positive. Alternatively, the individual might touch the target accidentally several times in the beginning of the stage and continue to do so, in which case the estimate for the intercept will be positive and significant. We decided to evaluate whether the individual achieved an understanding of the task in the current stage by means of the following approach: after fitting the model, we first determined, in link space, the probability of touching the target (and its standard error) at the very last touch (i.e., after potential learning took place). In link space, the likelihood function of the probability can be assumed to have the shape of a normal distribution with a mean of *log(pt/(1-pt))* and a standard deviation being the estimated standard error of *log(pt/(1-pt))*, whereby both *log(pt/(1-pt)*) and its standard error result from the fitted model and *pt* is the estimated probability to touch the target at the very last touch of the stage. Based on this normal distribution, we finally determined the probability of *pt* to be at chance level (i.e., 0.3; see below) or below for that given stage, and if this was less than or equal to 0.05, we considered that the individual had learned the rule of that stage. Note that this criterion corresponds to a Bayesian approach assuming flat priors and deciding that the individual had mastered the current stage if its performance is between 0.3 and 1 with a probability 95%. In that sense, one could also state that applying the above criterion means to be 95% confident that the individual had employed the contextual rule. In other words, when the probability of touching the target at the very last touch of a stage was larger than 0.3 (i.e., above chance) with 0.95 confidence, we considered the stage being solved by rule.

To clarify more in detail, we determined the chance probability for touching the target as follows: (1) the probability to touch the target at the first touch of a trial (pt1) is 1/4. To touch the target with the second touch of a trial (pt2), the individual first has to touch one of the two neutral stimuli and then the target. Hence, the probability to touch the target at the second touch is (2/4) *(1/3) and at the third touch of a trial (pt3) is (2/4) *(1/3) *(1/2). Analogously, the probabilities to touch the distractor with the first, second, and third touch (pd1, pd2, and pd3) are as the same as for the target. As a result, the overall chance probability to touch the target can be determined as the weighted sum of the number of target touches in each of the six possible outcomes, divided by the weighted sum of the total number of touches in each of the six possible outcomes: pt = (pt1*1 + pt2*1 + pt3*1 + pd1*0 + pd2*0 + pd3*0)/ (pt1*1 + pt2*2 + pt3*3 + pd1*1 + pd2*2 + pd3*3) =0.3. This was also confirmed by a simulation run with the same transition parameters used in the experiment.

### Second-step modeling

After the chance-level estimation we determined for each stage, at which point (touch) the probability of choosing the correct stimulus reached the value of 0.8. When this estimated touch number was equal or smaller than zero, we excluded the stage from further analysis, which resulted in the exclusion of 128 stages. We also excluded stages in which the estimated number of touches, needed to reach 0.8 probability of touching the target, was larger than the maximum number of touches across all sessions, which resulted in the exclusion of 120 stages. Finally, we excluded the last cycle of each session. The final data set comprised of 2,182 estimated number of touches, needed to reach a 0.8 probability to touch the target, which were then modeled as a function of cycle index (across the whole experiment), species (monkey or human), stage type (discrimination or reversal), feature dimension (color, motion, or shape), and training order (AUT1, AUT2 or AUT3). Given that the effect of cycle index could depend on the particular combination of the other four, we also included all interactions up to the fifth order into the model. In addition, the model included a random intercepts effect for the identity of the individual. Finally, to avoid an overconfident model, we included random slopes (Schielzeth and Forstmeier, 2009; Barr et al., 2013) of cycle index, stage type, and relevant feature as well as also all their interactions up to the third order. We could not include parameters for the correlations among random intercept and slopes as a respective model failed to converge. With the aim of evaluating all the fixed effects in the full model and avoid confounds deriving from multiple testing (Forstmeier and Schielzeth, 2011), we first compared the full model with a null model having the intercept and training type as fixed effects and a random effect part identical to that of the full model. If this comparison reveals significance, we can conclude that at least one of the terms (main effects or interactions) present in the full model but absent in the null model significantly affected subject performance.

### Statistical implementation

All statistical analyses were carried out in R (version 4.2.x; R Core Team, 2021). The *Chance-level estimation* models were fitted using the function glm. The *second-step modeling* consisted of a Generalized Linear Mixed Model (GLM; Baayen, 2008) with gamma error distribution (Bolker, 2008) and logit link function (McCullagh and Nelder, 1989). We fitted the model using the function *glmer* of the package *lme4* (version 1.1–29; Bates et al., 2015) with the optimizer “*loptwrap*.” Prior to fitting, we z-transformed cycle index to a mean of zero and a standard deviation of one to ease model convergence and achieve easier interpretable estimates (Schielzeth, 2010). In order to obtain value of ps for individual fixed effects in a given model, we dropped them, one at a time, and compared the simplified models with the given model (R-function *drop1*). All model comparisons utilized a likelihood ratio test (Dobson, 2002). We determined model stability by dropping individuals from the data set one at a time, fitting the full model to each of the subsets and finally comparing the derived estimates with those obtained for the full data set. This revealed the model to be of good stability (see Supplementary Material). We determined 95% confidence limits of model estimates and fitted values by mean of a parametric bootstrap (*N* = 1,000 bootstraps; function bootMer of the package lme4). The sample analyzed with this model comprised a total of 2,182 0.8 values (one per stage) for 36 individuals (25 humans and 11 monkeys). The response was not overdispersed given a model (dispersion parameter of 0.64).

### Cognitive flexibility index

In order to compare the cognitive flexibility across species, stimulus features, and types of shifts, we computed a cognitive flexibility index (CFI) for each participant, based on the stages solved by rule (see *chance-level estimation*):


(1) $$\text{Cognitive}\;\text{Flexibility}\;\text{Index}=\frac{\text{number}\;\text{of}\;\text{stages}}{\text{number}\;\text{of}\;\text{trials}}$$

Here the number of trials, considered to be a proxy for the number of attempts made at finding the stage’s rule, was normalized by the number of stages solved by rule (with an 80% confidence). Finally, we assessed statistically significant differences in CFIs between the different rule and shift types. Given that all the CFI distributions resulted to be non-normally distributed we used non-parametric tests. We used a Wilcoxon paired two-sided signed rank test to assess CFI differences across rule types (color, motion, shape) for humans and monkeys separately. To test difference between intra- and extra-dimensional shifts we used a non-paired rank sum test due to unequal number of samples of each of the two shifts for each individual. All p values were compared to an alpha level that was adjusted according to the number of tests run in each analysis.

## Results

**Figure 2:**
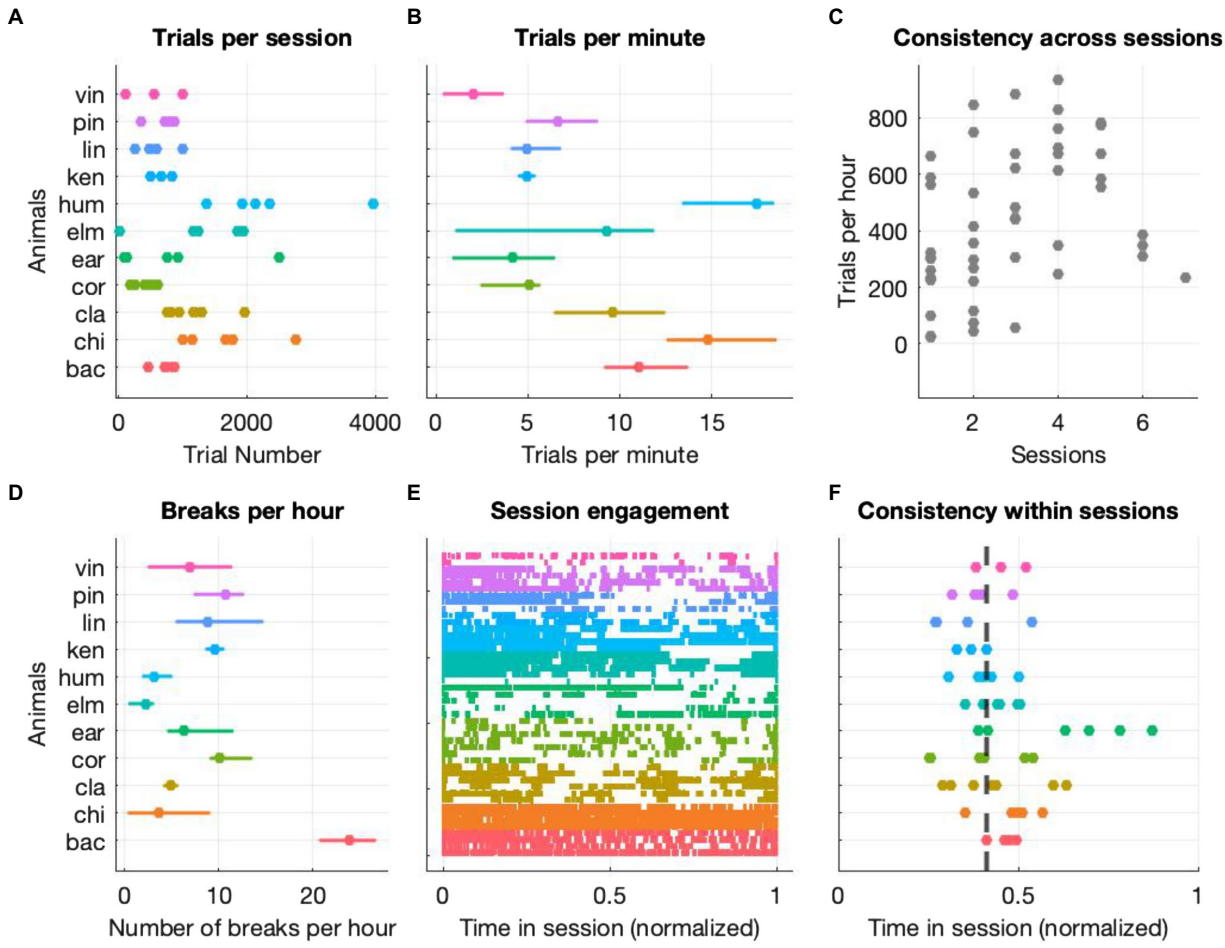
General level of engagement (monkeys only). **(A)** Number of trials performed by each animal with each data point representing one testing session **(B)** median number of trials per minute with variation across sessions expressed as interquartile range **(C)** average trials per hour for each session (across all animals) as function of consecutive sessions **(D)** median number of breaks per hour with variation across sessions expressed as interquartile range **(E)** event plot of all trials with each color representing one animal and each row representing a single session. Time is normalized to the session end. **(F)** Each dot represents the median trial time (normalized to the session end) of each session and animal. For each trial of each session, we first normalized all timestamps of each trial start to the session duration and then computed the median of the resulting distribution. Finally, we computed the median of all the medians and found that across all animals and sessions the median time point at which half of the trials were performed was 0.41 of the session’s duration, the dashed line on the figure.

We assessed cognitive flexibility of 26 participants (25 humans and 11 macaque monkeys) with a custom-made, touchscreen-based task that combines design elements of classical reversal-learning and set-shifting procedures. In order to assess cognitive flexibility of humans and monkeys on a non-binary set of rules, we employed motion along with color and shape as feature dimensions. Given that motion has never been used in this context before, we also structured our task in a way that rule changes would automatically occur after repeated interactions at chance level. This was instrumental to prevent participants (especially animals) from being stuck at potentially difficult rules. We started by determining the animals’ level of general engagement in the experimental sessions. We looked at number of trials per session and per minute, the time at which the most engagement took place and the number and length of breaks (defined as an inter-trial-interval of more than 30 s), and included all stages regardless of them being completed following the rule or by chance. Figure 2 depicts the animals’ level of general engagement to be taken as a description of the conditions under which the cognitive assessment was carried out. Although the levels of interaction, as quantified by the number of trials performed per session (Figure 2A) and per minute (Figure 2B) and the number of breaks per hour (Figure 2D) were variable across animals, each animal had a consistent interaction level within and across sessions (Figures 2C,E,F). We have performed a similar analysis for humans to determine their general engagement level as well (Supplementary Figure S2).

**Figure 3:**
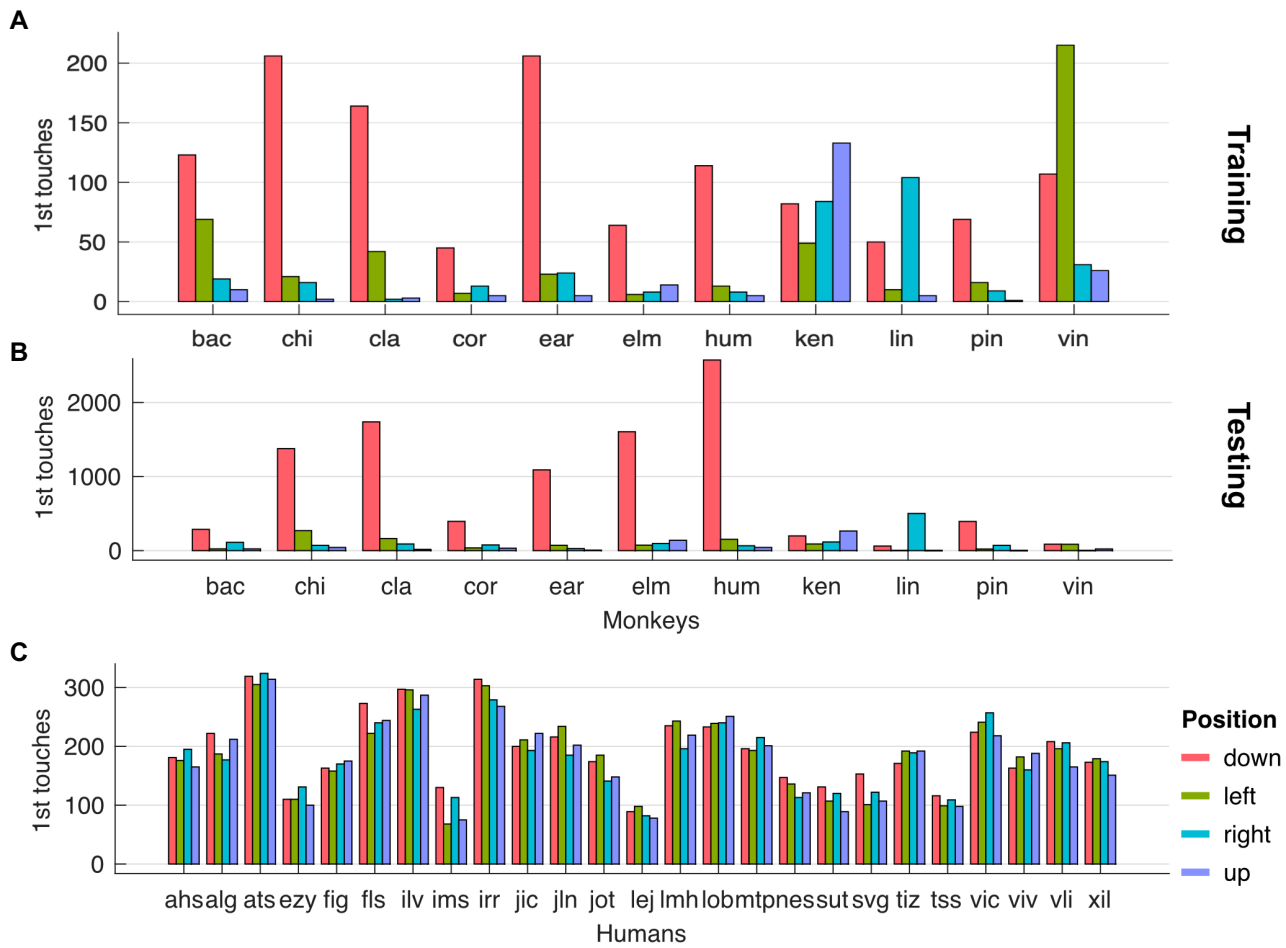
**(A)** Side bias for monkeys in testing stages. **(B)** Side bias for humans in testing stages. (**C)** Side bias for monkeys in training stages.

We observed a strong side bias towards the stimulus located at the bottom of the screen, for monkeys only, across testing (Figure 3A) and training stages (Figure 3C). We computed the side bias by counting the occurrences of the first touch of each trial with respect to the stimulus location (up, down, left and right). Finally, we found no correlation between the strength of side bias and the magnitude of animals’ flexibility.

**Figure 4:**
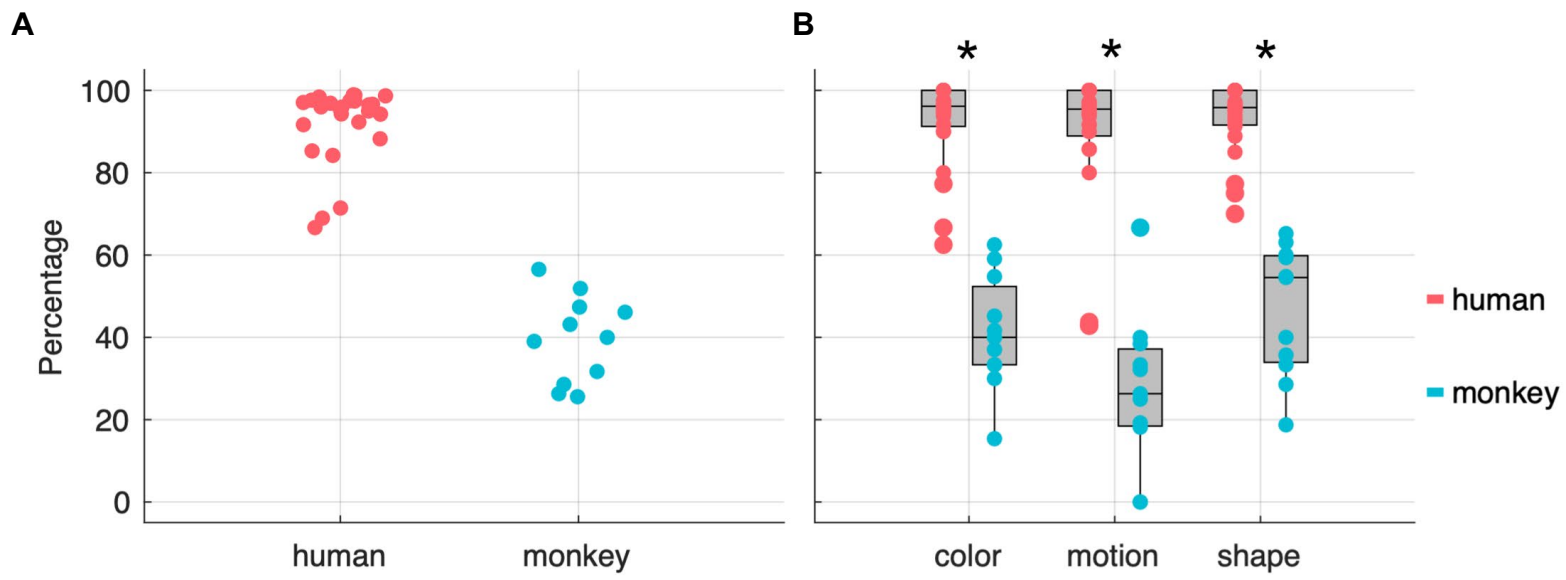
Percentages of stages solved by rule – at an 80% confidence threshold – across both species **(A)** and feature dimensions **(B)**. The stars indicate statistical significance at a paired Wilcoxon rank sum test.

Moreover, the *second-step modeling* revealed a clearly significant full-null model comparison (χ^2^ = 185.6, df = 69, *p* < 0.001) indicating that cycle number and/or species (monkey or human) and/or stage (discrimination or reversal) and/or feature (color, direction, or shape) and/or any of the interactions between them or with training type significantly contributed to the response. Moreover, the model revealed a clear difference between species, whereby monkeys needed more trials than humans to reach a 0.8 probability of choosing the correct stimulus (151 touches on average for monkeys and 5 for humans; median of medians per individual - see Supplementary Table S1; Supplementary Figure S3). Finally, the effect of cycle number on the number of touches needed to reach the 0.8 threshold varied depending on the particular combination of species, stage type (discrimination or reversal), relevant feature, and training type. However, given that such effects were usually associated with considerable uncertainty, these results should be taken only as proof of concept that with such tasks it is possible to trigger reversal-learning as well as set-shifting effects. More information and visualizations about the results from the *second-step modeling* are available in the Supplementary material. In order to quantify cognitive flexibility, we focused only on those stages which were solved by following the rule, ignoring those that were solved by chance. To achieve this, we used the probability values from the *chance-level estimation* analysis (see *Data Analysis* in Materials and Methods) to separate the two. With this modeling analysis we assessed the evolution of trials’ outcome to find the likelihood of such sequence being produced by a stochastic process or by the participants knowledge of the contextual rule. As a result, we quantified whether, and in how many stages, participants performed differently than chance. Figure 4A shows the percentages of stages for which there is at least an 80% probability that they were completed by rule. We decided to use an 80% threshold in order to be conservative in filtering out pure chance stages, but not too strict to remove all stages that were solved by a combination of chance and rule. In Figure 4B we further separated the rule-based stages by the three task-relevant features (color, motion, and shape). We found that the proportion of rule-based stages between the two species was significantly different (Wilcoxon rank sum test, for color: test = 599.5, *p* = 2.52E+08; for motion: test = 597, *p* = 3.68E+08; for shape: test = 600; *p* = 2.24E+08; all with *N* = 36, 25 humans vs. 11 monkeys; and corrected to an alpha level of 0.017) but not significantly different within individuals of the same species (paired, two-sided, Wilcoxon rank sum test), suggesting that from the participants point of view the three features provided equivalent cognitive challenges. Note that we have also run a simulation with a stochastic agent for 100,000 simulated stages (Supplementary Figure S5) and found that while humans and monkeys performed a median of 10 and 41 trials respectively before the rule spontaneously changed, in this simulation the rule changed after 50 trials, i.e., it took 50 trials for the agent to complete a stage. However, we believe that the modeling approach described in the Methods section captures a fairer representation of the participants’ behavior. The modeling is indeed able to better disentangle stages comprised exclusively by random trials from stages where participants interacted randomly at the beginning of the stage and purposefully towards the end.

**Figure 5:**
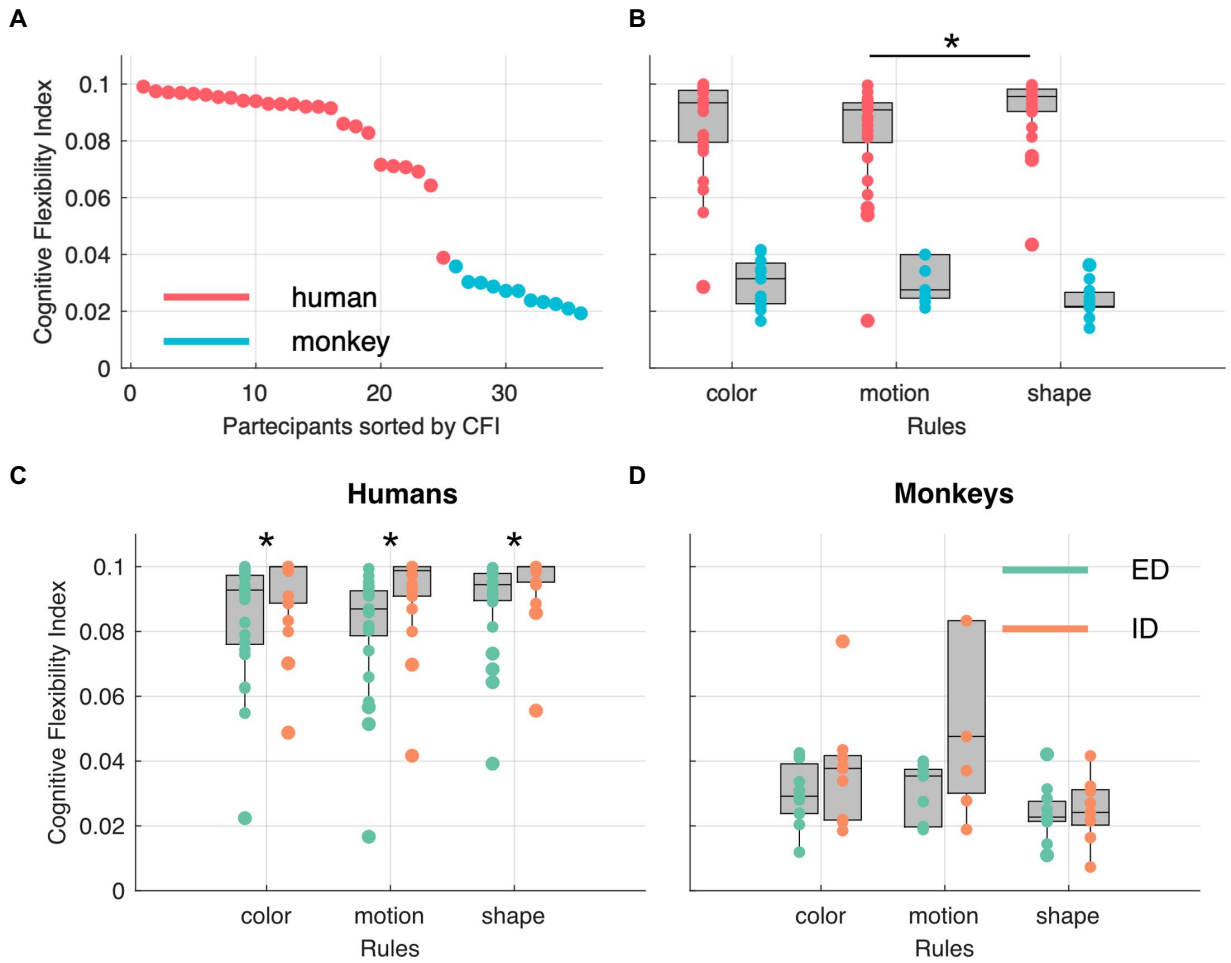
Participants sorted by cognitive flexibility index (CFI) across species **(A)** and rules **(B)**. Star indicates statistical significance at a paired Wilcoxon rank sum test, with an alpha level of 0.0083 (corrected for multiple comparisons). **(C,D)** show CFI as function of rules in intra-dimensional (ID) and extra-dimensional (ED) shifts. Stars indicate statistical significance at a Wilcoxon rank sum, non-paired, two-sided test.

With the cognitive flexibility index (*see cognitive flexibility index – CFI* in Materials and Methods) based only on the stages solved mostly by rule, we compared performances across species, contextual rules, and shift types. In Figure 5A all participants are sorted according to their respective CFI and, as expected, the two species are clearly separated from one another. In Figure 5B, the CFI values are represented as a function of feature dimension. We observed a significantly higher CFI in humans in shape stages compared to motion stages and no other statistically significant differences across the three features and the two species was observed (see Table 1). Finally, we investigated whether our paradigm, and relative flexibility index, could replicate known differences between intra-(ID) and extra-dimensional (ED) rule shifts. To test this hypothesis, we computed the CFIs separately for each species and each feature. While humans showed significantly higher CFIs for intra-dimensional shifts compared to extra-dimensional ones (see Table 2) we observed no significant differences in ID and ED in monkeys. Finally, note that in order to perform this analysis, both the humans’ and the monkeys’ dataset were split in two, one with ED shifts and one with the ID shifts. In particular in the monkeys’ dataset, this resulted in small sample sizes.

**Table 1:** Overview of the results of the Wilcoxon paired, two-sided, signed rank test on CFI differences across rule types (color, motion, and shape) for humans and monkeys separately (Figure 5B).

Species	Comparison	Test statics	Value of *p*	*N*	*p* < 0.05/6
Humans	Color/motion	241	0.0347	25	n.s.
	Color/shape	104	0.1155		n.s.
	Motion/shape	21	0.0001		sig.
Monkeys	Color/motion	18	0.6523	9	n.s.
	Color/shape	35	0.1641		n.s.
	Motion/shape	39	0.0547		n.s.

Significant differences are denoted by sig. in the last column in which the value of *p* of each comparison is compared to an alpha level that is adjusted for multiple comparisons (0.008).

**Table 2:** Overview of the results from the Wilcoxon rank sum, non-paired, two-sided test on CFI differences between intra-dimensional and extra-dimensional shifts, separately for rule types (color, motion, shape) and humans and monkeys separately (Figures 5C,D).

Species	ID/ED comparison	Test statics	Value of *p*	N of ID	N of ED	<0.05/6
Humans	color	575	0.0081	20	25	Sig.
	motion	693	0.0004	22	25	Sig.
	shape	699	0.0002	22	25	Sig
Monkeys	color	105	0.4474	9	11	n.s.
	motion	75	0.1056	7	9	n.s.
	shape	98	0.8194	9	11	n.s.

Significant differences are denoted by sig. in the last column in which the value of *p* of each comparison is compared to an alpha level that is adjusted for multiple comparisons (0.008).

## Discussion

Set-shifting and reversal learning paradigms have been extensively used in neuroscience and psychology. Across species, they have allowed an efficient characterization of cognitive flexibility of different populations, including clinical ones (Waltz and Gold, 2007; McKirdy et al., 2009; Remijnse et al., 2009; Izquierdo and Jentsch, 2012; Reddy et al., 2016). They have also been used to assess the psychological wellbeing of non-human primates (Judge et al., 2011; Pomerantz et al., 2012). However, while reversal learning has been used with both humans and non-human animals, most set-shifting paradigms do not generalize well to non-human animals (see Uddin, 2021 for a review). In order to evaluate various aspects of cognitive flexibility from a comparative perspective we developed a task that contains both set-shifting and reversal learning features. Similar to more established set-shifting procedures (e.g., Wisconsin Card Sorting Test – WCST) we used four visual stimuli made from four unique combinations of three visual features: shape, color, direction of visual motion, each with four categories (e.g., upward, downward, rightward, and leftward motion). On the other hand, we structured the trials with a reversal-learning-like approach in mind, by assigning contextual rules as rewarded and unrewarded to two categories of a given feature (discrimination phase) and reversing the rule once the discrimination was learned (reversal phase). With this unified approach we were able to assess how both monkeys and humans switched between multiple rules. We here report the cognitive flexibility of both humans and rhesus macaques, assessed with such a unified approach.

### Use of motion as an additional visual dimension for cognitive tasks

We included motion as a feature dimension (in addition to the commonly used shape and color) to be able to assess non-binary rule switches. Visual motion information is processed in a very similar way as color and shape in the primate brain (Self and Zeki, 2005; Handa et al., 2010; Van Essen et al., 2019) and thus provides a more compatible feature dimension when combined with color and shape, compared to the use of quantity (Grant and Berg, 1948; Nelson, 1976; Stuss et al., 2000; Prentice et al., 2008; Nyhus and Barceló, 2009; Lange et al., 2018; D’Alessandro et al., 2020; Uddin, 2021). Quantity might not only be particularly difficult to infer for animals in general and non-human primates in particular (Nieder, 2020), but it might also require substantially different types of computation and relatively higher cognitive effort and competences, compared to color and shape (Dias et al., 1996a; Nagahama, 2005; Kuwabara et al., 2014). Motion, on the other hand, seems more appropriately comparable to color and shape, as motion has been shown (1) to be easily detected and discriminated by NHPs and (2) to be processed early along the visual hierarchy. We found that while participants were able to find and use motion rules as well as shape and color rules, suggesting that motion provides a cognitive challenge comparable to color and shape, they did so with variable and significantly different levels of efficiency, across species, features, and shift types.

### Foraging-like approach to potentially minimize frustration

Given that, to the best of our knowledge, this is the first time that a set-shifting task with three rules is used with NHPs and that our animals were fluid controlled only for the duration of the session, it is reasonable to expect animals to refrain from interacting when the task becomes too difficult. We adopted two main measures to potentially minimize such frustration in favor of a more pleasant engagement. First, in order to avoid animals being stuck with certain contextual rules, we allowed the rule to spontaneously change after a certain number of interactions. In this study, the likelihood of the rule to spontaneously change after 80 trials was around 80%. With this approach animals that faced difficulties to understand the rule (or that had a momentary lapse in motivation to seek the rule) were given another chance with a new rule. During data analysis the stages aided by this algorithm were quantified and excluded from the calculation of the cognitive flexibility index. Second, with every trial we presented four stimuli, two of which were distractor stimuli (*neutrals*) that did not terminate the trial when selected, but simply disappeared. This was instrumental to allow the animals to adopt a foraging-like strategy in which making a wrong choice (1) does not lead to a reward but (2) does not prevent the animal from reaching it within the same trial and (3) it might provide additional help to obtain the reward. While a description of the specific strategies in the use of such neutral stimuli is beyond the scope of this paper, we believe that this aspect is at the core of the sustained engagement level we observed across most of our animals. On the other hand, it has to be noted that all the animals, but none of the humans, quickly developed a strong bias towards the stimulus closer to the bottom of the screen, which was selected first in over 50% of the trials. Unfortunately, given that the trial start button was also positioned at a location overlapping with the position of the bottom stimulus, it is not entirely clear whether such bias resulted mainly from a strategy or a design choice. Future experiments, with fewer and/or a different stimulus configuration, will likely be able to disentangle these two possibilities.

### General engagement level with cage-based cognitive assessment in monkeys

We observe a sustained level of engagement within and between sessions across all animals. As animals were provided with *ad libitum* water and food after each session, we argue that such engagement might not be solely attributable to the sugary fluid the animals received as reward. We instead argue that such high engagement is partly due to the self-paced nature of our assessment. Animals could choose how and when to interact with the device and when to take breaks to perform other activities or simply detach from the task altogether. On the other hand, 2 out of 14 animals did not interact with the device enough to complete the training procedure, despite ample access. Finally, before the assessment itself, some form of automated training procedure was necessary to bring all the animals to the same level of understanding (Berger et al., 2018; Calapai et al., 2022). Overall, the entire procedure is feasible as an opportunistic testing routine, i.e., to be carried out in the animal’s downtime from other experimental procedures.

### Intra-dimensional and extra-dimensional shifts with more than two rules

As a proof of concept, we provide a quantification of our participants across different types of set-shifting behaviors, namely intra- and extra-dimensional shifts (Shnitko et al., 2017; Ciampoli et al., 2021; Weiss et al., 2021), known to be associated with different areas of the frontal cortex in primates (Dias et al., 1996b; Hampshire and Owen, 2006; Robbins, 2007). To the best of our knowledge, studies with non-human primates in which intra- and extra-dimensional shifts are compared with each other, make use of paradigms in which it is not possible to dissociate a rule switch from a reversal (Shnitko et al., 2017). This is due to the binary nature of most paradigms developed for animals that often present two types of stimuli (rewarded or unrewarded), two possible rules (mostly shape and color), and two categories from such rule. When compared to set-shifting paradigms developed for humans, in which multiple rules and multiple objects are presented at every trial, results obtained from animal versions of these tasks are difficult to generalize to humans. Analogously, comparing shifts within the current contextual rule to shifts from different rules is problematic when only binary choices are available. In the case of intra- vs. extra-dimensional shifts, for example, each category (e.g., blue and red) and each feature (e.g., shape and color), negate each other, making the process of rule searching quicker and more efficient compared to a non-binary scenario. Finding that the blue stimulus is not rewarded anymore, for example, not only means that the category blue is now the non-rewarded one, but implicitly suggests that the red stimulus is now the correct one. By extension, if the color rule is not the relevant one, then the shape must be the one. In a non-binary scenario, like in our paradigm, if the relevant rule or category is not relevant anymore, the participants are required to search for more evidence. Thus, with the increased number of attempts required in non-binary paradigms, the nature of such attempts truly differentiates a rule switch from a rule flip in general and intra- vs. extra-dimensional shifts in particular. We have observed a discrepancy between humans and monkeys when we compared their performance for intra- and extra-dimensional shifts. While humans performed better during intra-dimensional shifts compared to extra-dimensional shifts for each and every feature/contextual rule (Figure 5C), monkeys showed no difference between the shifts (Figure 5D). We believe that this might be because of the different types of approaches humans and monkeys use to find the rule. Humans might be using the two strategies we mentioned above: rule flip and rule switch, at different times during the task. For instance, they might be doing a rule flip when the reward contingencies of target and distractor are flipped upon reversal but doing a rule switch when a cycle (discrimination-reversal pair) is completed and another cycle with a new contextual rule starts. On the other hand, monkeys might be performing similarly for intra- and extra-dimensional shifts as they are potentially switching the rule all the time and considering each and every stage as novel even if it is a reversal stage and thus the reward contingencies are flipped only. While this is merely speculative, this difference between animals and humans in operating one or multiple strategies to change rule is in itself a sign of significantly higher cognitive flexibility of humans over monkeys.

### Limitations of the study

Our paradigm combines a reversal-learning-like structure (namely a succession of discrimination and reversal blocks) with a set-shifting-like configuration of stimuli and contextual rules (namely presenting four stimuli and three possible rules at every trial). Our aim was to unify these paradigms into a single non-binary set-shifting paradigm that can be used across different species of primates, including humans with different types of rule shifts, like intra-dimensional vs. extra-dimensional shifts, as well as various switch costs (Altmann, 2007; Rayburn-Reeves et al., 2011, 2013, 2017). While we were able to assess, compare, and characterize most of these aspects, we could not reliably measure the switch cost at the reversal point in monkeys (one of the hallmark signatures of typical reversal-learning paradigms). The switch cost is typically intended as the cost (in terms of number of attempts required) that participants pay when a rule is reversed unbeknownst to them. In classical reversal learning tasks switch costs decrease as a function of attempts, choices, or trials, and it is used as a proxy for the ability of a given subject to disengage from an acquired rule. While we could indeed observe switch costs in our humans, most of our animals showed patterns of responses that we deemed to be too noisy for a reliable quantification.

Incidentally, we also observed a strong preference, across most animals, for the stimulus at the very bottom of the screen. This behavior might had been triggered by the position of the trial start button, which monkeys needed to touch to initiate each trial. Even though the start trial and the bottom stimulus never overlapped in time, they largely did so in space. From the perspective of the monkeys, it seems reasonable to assume that selecting this stimulus constituted substantially less effort than selecting the others, placed far away from the location of the trial start button. This likely resulted in an inflated number of choices of bottom stimuli in the animal data that made a quantification of the switch cost unreliable. We believe that both these issues are related to the foraging-like architecture of the paradigm. While the side bias could very well be a foraging strategy rather than an experimental issue; we here nonetheless provide suggestions that could alleviate both issues at once. First, a better positioning of the stimuli could prevent animals from developing effort-related foraging strategies. Such balancing of the effort required to reach each stimulus could be achieved by showing the stimuli in a radius that is equidistant from the trial start. Second, reducing the number of options (namely of stimuli on the screen) from four to three could reduce the task-related cognitive overload. As a consequence, animals could have more resources to focus on finding the contextual rule from the trial start. Third, a stricter threshold to advance to the next rule (in our study: 80% hit rate across the last 10 trials) could be used as an incentive for the animals to engage less casually with the task. Nonetheless, we advise caution when increasing the difficulty of such tasks when conducting self-paced cognitive assessment (or training) of captive animals with limited fluid control. Animals might indeed assume that the difficulty level does not justify their effort and decide to stop engaging altogether.

## Conclusion

In conclusion, we assessed cognitive flexibility of adult humans and captive rhesus macaques by using a novel set-shifting paradigm with three visual dimensions (shape, color, and motion) and with a foraging approach (neutral stimuli and chance-aided advancement). We found that monkeys engaged with the task voluntarily despite having minimal dietary restriction and that humans and monkeys could both use motion information as flexibly as shape and color information across extra-dimensional and intra-dimensional shifts. Due to a side bias observed across the vast majority of animals, we could not reliably quantify switch costs in monkeys. Our study shows that the motion direction of a visual stimulus on a screen can be easily and effectively used by both monkeys and humans as a feature dimension in a complex cognitive task. This is especially relevant when assessing set-shifting abilities of both species beyond the classical shape-color dichotomy. With such an approach, aspects from common reversal-learning approaches and set-shifting approaches can be combined and quantified within a unified paradigm.

## Data availability statement

The datasets presented in this study can be found in online repositories. The names of the repository/repositories and accession number(s) can be found at: https://doi.org/10.25625/X2SK2K.

## Ethics statement

Ethical review and approval was not required for the study on human participants in accordance with the local legislation and institutional requirements. The patients/participants provided their written informed consent to participate in this study. The animal study was reviewed and approved by Niedersaechsisches Landesamt für Verbraucherschutz und Lebensmittelsicherheit.

## Author contributions

PY, AC, and ST conceptualized the study. PY collected the data. AC curated the data. PY and AC analyzed the data and wrote the manuscript with input from ST. RM conceptualized and conducted the statistical analysis. RM wrote statistical descriptions. ST provided funding for the study. All authors discussed and interpreted the results.

## Funding

This work was funded by the Cognitive Neuroscience Laboratory of the German Primate Center.

## Conflict of interest

The authors declare that the research was conducted in the absence of any commercial or financial relationships that could be construed as a potential conflict of interest.

## Publisher’s note

All claims expressed in this article are solely those of the authors and do not necessarily represent those of their affiliated organizations, or those of the publisher, the editors and the reviewers. Any product that may be evaluated in this article, or claim that may be made by its manufacturer, is not guaranteed or endorsed by the publisher.

## Footnotes

^1^ http://mworks-project.org
