# Supplementary material for: Assessing cognitive flexibility in humans and rhesus macaques with visual motion and neutral distractors
Source: Front Psychol. 2022 Dec 20;13:1047292. doi: 10.3389/fpsyg.2022.1047292 (PMC9807625; doi:10.3389/fpsyg.2022.1047292)
Supplement: Supplementary file 1 [file Data_Sheet_1.pdf]

*Supplementary information for*

# Assessing cognitive flexibility in humans and rhesus macaques with visual motion and neutral stimuli

**Yurt, Pinar*[1,3]*; Calapai, Antonino*[1,2*]*; Roger Mundry*[2,4,5]*; Treue, Stefan*[1,2]***
[1]Cognitive Neuroscience Laboratory, German Primate Center, Goettingen, Germany;
[2]LeibnizScienceCampus Primate Cognition, Goettingen, Germany;
[3]Georg-August University School of Science, Goettingen, Germany;
[4]Cognitive Ethology Laboratory, German Primate Center, Leibniz Institute for Primate Research, Kellnerweg 4, 37077 Goettingen, Germany;
[5]Department for Primate Cognition, Georg-August-University Goettingen, Germany;

*** Correspondence:**
Corresponding Author
ACalapai@dpz.eu

**Supplementary Figure 1 –** Switch cost across monkeys and humans

**Supplementary Figure 2 –** General engagement level of human participants

**Supplementary Figure 3 –** Effects of all five predictors of the second-step model

**Supplementary Figure 4 –** The data and the fitted models

**Supplementary Figure 5 -** Comparison of number of trials between a simulated agent, humans and monkeys

**Supplementary Table 1 –** Results of the full second-step model

**Supplementary Table 2 –** Descriptive information on monkeys and their task performance

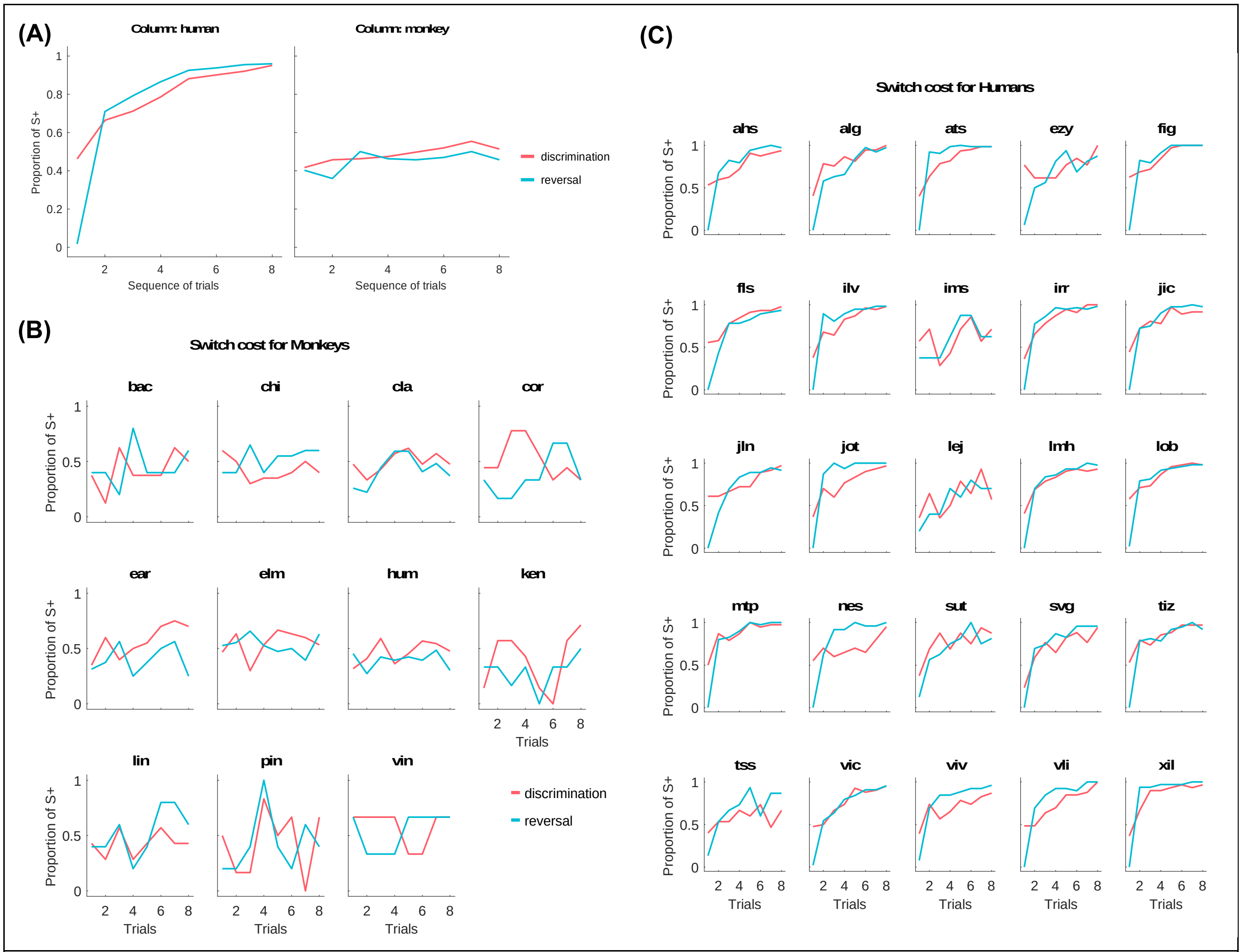


**Supplementary Figure 1.** Switch cost across monkeys and humans. **(A)** switch cost, monkeys and humans pooled separately, **(B)** switch cost for individual monkeys, **(C)** switch cost for individual humans. For the analysis, we determined the proportion of S+ (target) touches over the trials pooled across humans (A, left panel) and across monkeys (A, right panel). Although we observe that humans choose the target stimulus in respective discrimination (red) and reversal (blue) stages more often across the trials, it stays at around the same level for monkeys.

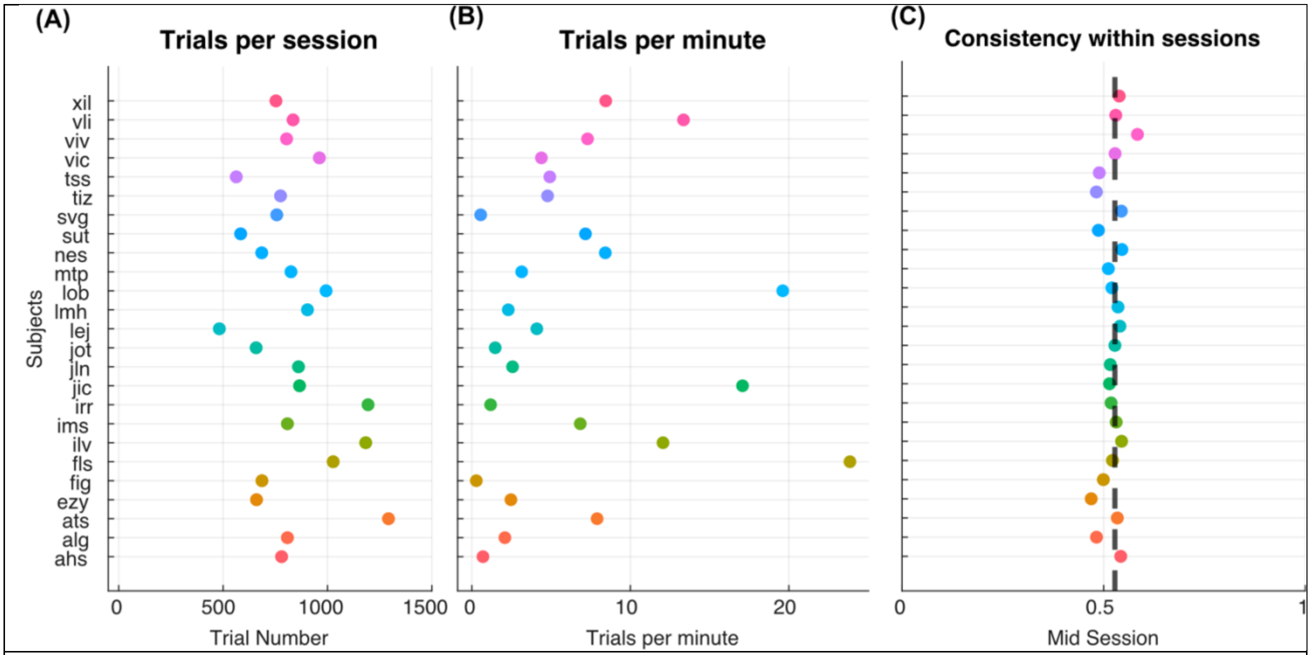


**Supplementary Figure 2.** General engagement level of human participants. We analyzed the general engagement level of humans in terms of the number of trials performed at each testing session **(A)**, number of trials performed per minute **(B)** and consistency within sessions **(C)**. In **(A)**, there is a single dot for each participant as they were tested in a single session. The dots in **(B)** represent the median of the session, which are color coded as in A. **(C)** Consistency within sessions i.e., how much participants interact in the first and second half of the sessions. Each dot represents a session and color coding is the same as in A and B. The dashed vertical line shows the median across all participants. Consistency within sessions is measured as in Figure 3 and these results show that humans consistently interacted in the first and the second halves of the sessions.

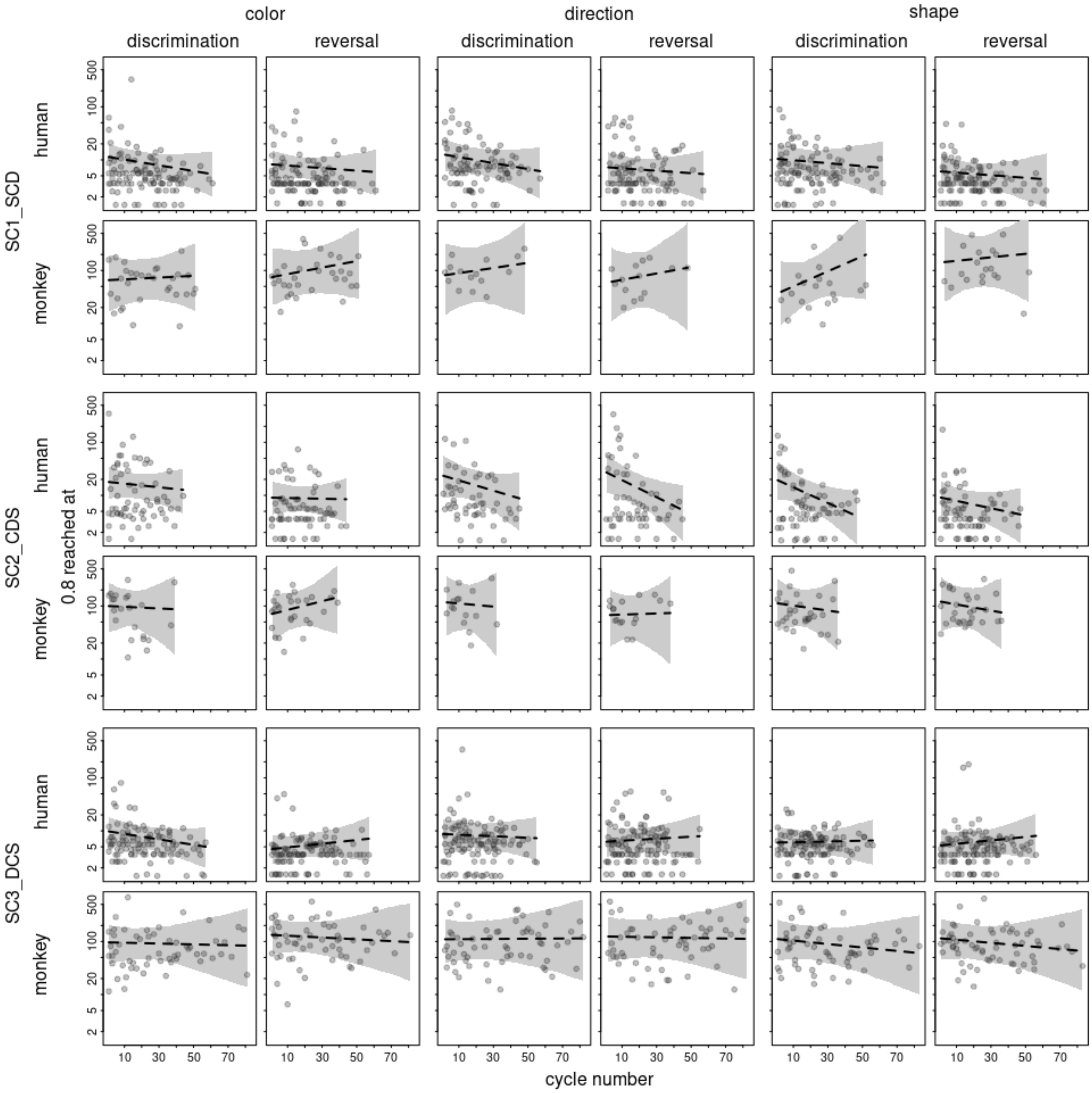


**Supplementary Figure 3.** Effects of all five predictors (as estimated with the full model) on at which touch a 0.8 probability of choosing the target was reached. Each dot shows an individual observation, and the dashed lines and grey polygons depict the fitted full model and its 95% confidence limits. Vertical blocks of two rows depict the three training types, and within each training type, humans appear in the top and monkeys in the bottom row. Horizontal blocks of columns depict the feature tested, and within features the discrimination and training phases appear on the left and right sides, respectively. Note that the depicted model comprises of all interactions up to the fifth order, but the five-way interaction is not significant (p=0.105).

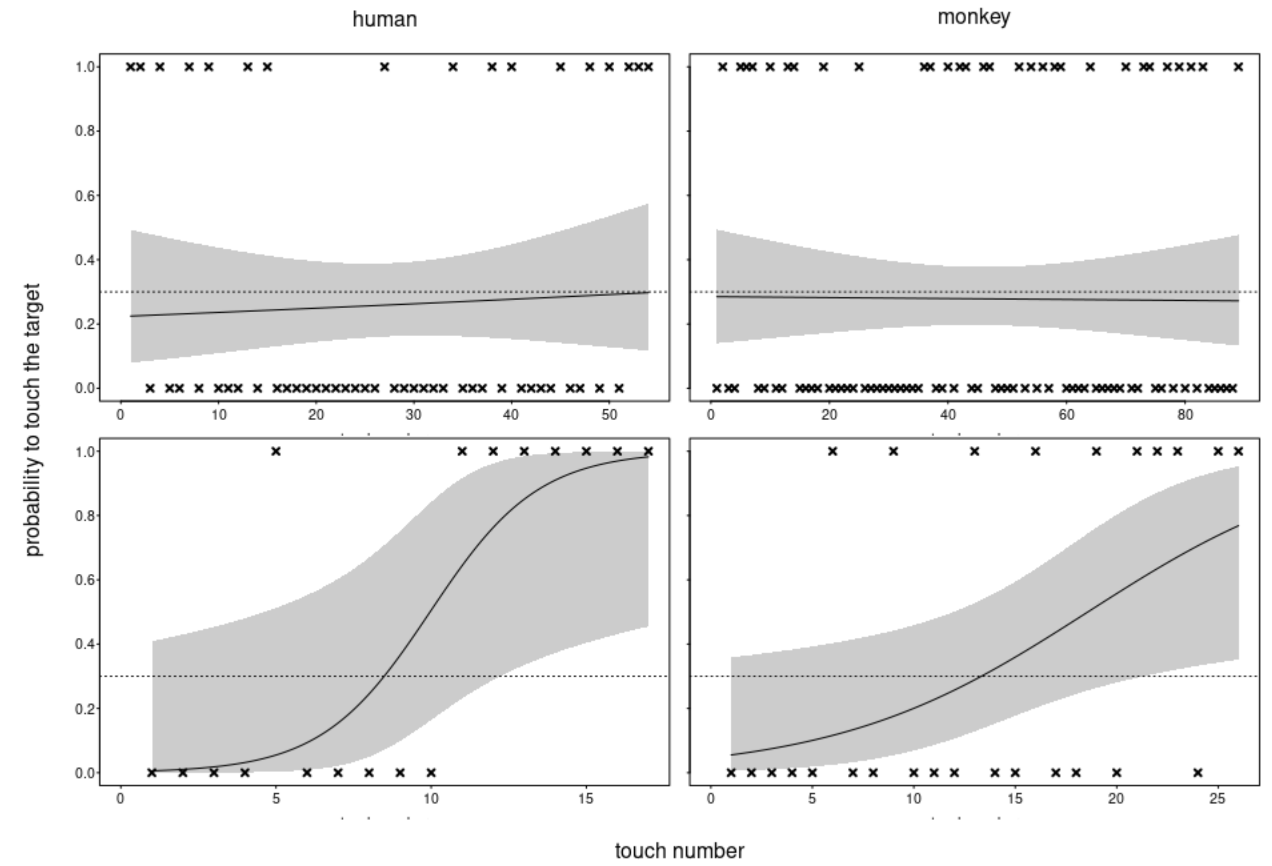


**Supplementary Figure 4.** The data and the fitted models for a human (left column) and a monkey (right column) who performed at random (top row) or learned to master the task (bottom row). Crosses show the individual touches (to the target or not) and black solid lines with surrounding grey polygons depict the fitted model and its 95% confidence interval.

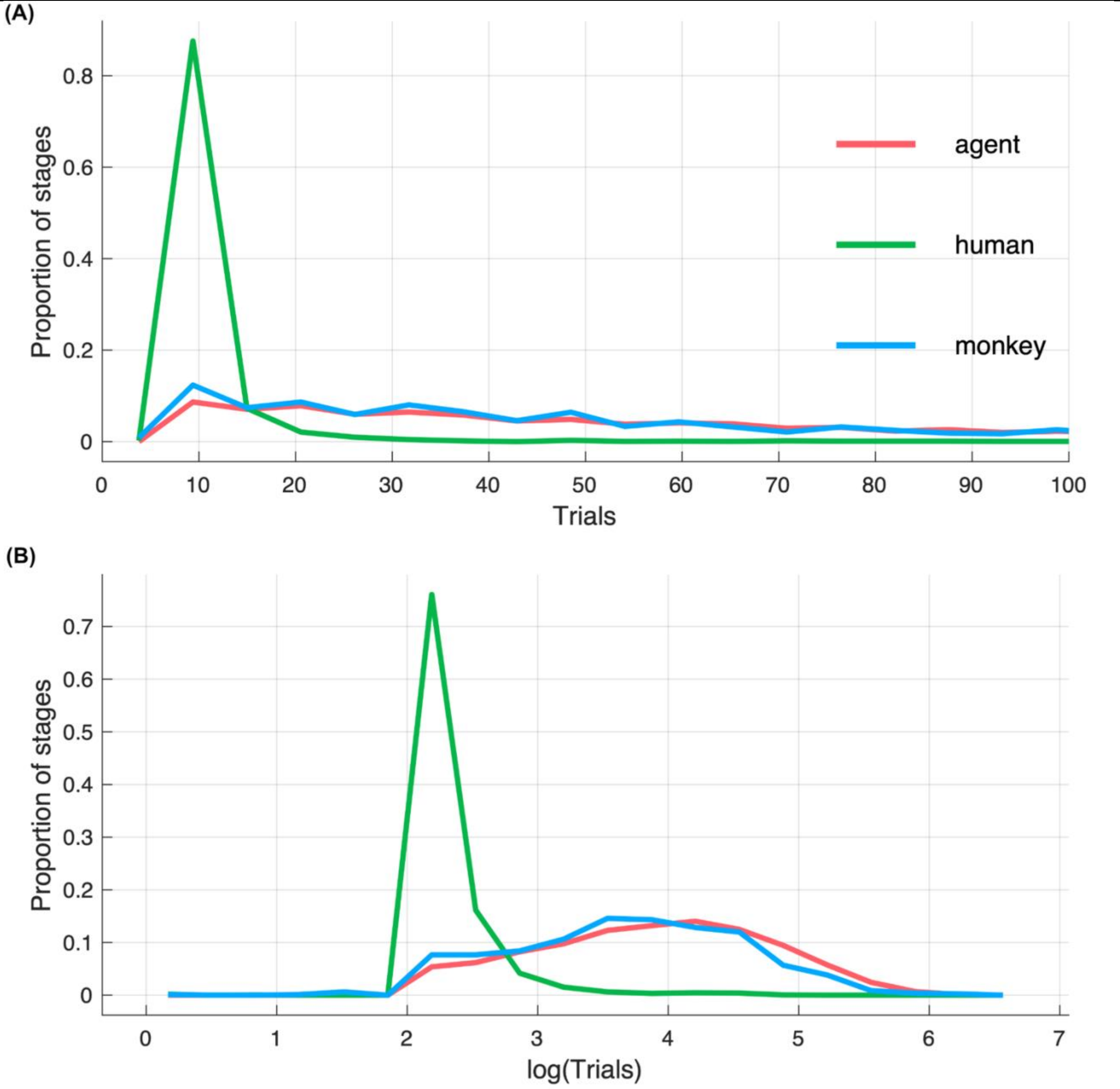


**Supplementary Figure 5.** Comparison of number of trials (**(A)** in absolute numbers **(B)** transformed to logarithmic scale) needed to complete each stage, between a simulated agent (red), human participants (green) and monkeys (blue). The agent consists of 100000 simulated stages generated following the same rules described in the *Methods – The experimental task*.

| term[1] | Estimate | SE | $Cl_{lower}$ | $Cl_{upper}$ | min | max |
|---|---|---|---|---|---|---|
| (Intercept) | 2.308 | 0.280 | 1.726 | 2.766 | 2.072 | 2.436 |
| z.cycle_counter | -0.279 | 0.147 | -0.562 | 0.013 | -0.311 | -0.179 |
| subjTypemonkey | 2.712 | 0.643 | 1.824 | 3.518 | 2.505 | 2.951 |
| stagereversal | -0.237 | 0.161 | -0.600 | 0.176 | -0.335 | -0.171 |
| featuredirection | 0.311 | 0.232 | -0.160 | 0.743 | 0.224 | 0.586 |
| featureshape | -0.026 | 0.274 | -0.543 | 0.505 | -0.175 | 0.220 |
| trainingTypeSC2_CDS | 0.680 | 0.426 | -0.118 | 1.411 | 0.457 | 0.895 |
| trainingTypeSC3_DCS | -0.150 | 0.389 | -0.959 | 0.586 | -0.360 | 0.081 |
| z.cycle_counter:subjTypemonkey | 0.212 | 0.295 | -0.406 | 0.776 | 0.099 | 0.382 |
| z.cycle_counter:stagereversal | 0.181 | 0.206 | -0.202 | 0.533 | 0.005 | 0.299 |
| subjTypemonkey:stagereversal | 0.615 | 0.343 | -0.176 | 1.383 | 0.546 | 0.837 |
| z.cycle_counter:featuredirection | 0.089 | 0.265 | -0.465 | 0.634 | -0.170 | 0.303 |
| z.cycle_counter:featureshape | -0.050 | 0.204 | -0.455 | 0.356 | -0.116 | 0.037 |
| subjTypemonkey:featuredirection | 0.023 | 0.537 | -0.609 | 0.505 | -0.239 | 0.222 |
| subjTypemonkey:featureshape | -0.365 | 0.627 | -1.338 | 0.652 | -0.614 | -0.238 |
| stagereversal:featuredirection | -0.164 | 0.270 | -0.777 | 0.424 | -0.349 | -0.014 |
| stagereversal:featureshape | -0.378 | 0.221 | -0.929 | 0.149 | -0.462 | -0.252 |
| z.cycle_counter:trainingTypeSC2_CDS | 0.514 | 0.234 | -0.046 | 1.060 | 0.365 | 0.612 |
| z.cycle_counter:trainingTypeSC3_DCS | 0.036 | 0.182 | -0.374 | 0.469 | -0.071 | 0.094 |

| | | | | | | |
|---|---|---|---|---|---|---|
| subjTypemonkey:trainingTypeSC2_CDS | -0.009 | 0.847 | -0.105 | 0.077 | -0.249 | 0.214 |
| subjTypemonkey:trainingTypeSC3_DCS | 0.174 | 0.797 | -1.177 | 1.520 | -0.061 | 0.390 |
| stagereversal:trainingTypeSC2_CDS | -0.324 | 0.264 | -0.961 | 0.303 | -0.430 | -0.128 |
| stagereversal:trainingTypeSC3_DCS | -0.238 | 0.211 | -0.796 | 0.348 | -0.294 | -0.143 |
| featuredirection:trainingTypeSC2_CDS | 0.063 | 0.362 | -0.720 | 0.874 | -0.167 | 0.237 |
| featureshape:trainingTypeSC2_CDS | -0.837 | 0.420 | -1.674 | -0.042 | -1.055 | -0.546 |
| featuredirection:trainingTypeSC3_DCS | -0.260 | 0.313 | -0.936 | 0.422 | -0.527 | -0.157 |
| featureshape:trainingTypeSC3_DCS | -0.295 | 0.372 | -1.088 | 0.514 | -0.543 | -0.141 |
| z.cycle_counter:subjTypemonkey:stagereversal | 0.125 | 0.427 | -0.679 | 0.917 | -0.325 | 0.268 |
| z.cycle_counter:subjTypemonkey:featuredirection | 0.253 | 0.612 | -1.085 | 1.601 | 0.008 | 0.526 |
| z.cycle_counter:subjTypemonkey:featureshape | -0.181 | 0.504 | -1.090 | 0.777 | -0.263 | -0.066 |
| z.cycle_counter:stagereversal:featuredirection | 0.128 | 0.353 | -0.605 | 0.875 | -0.132 | 0.309 |
| z.cycle_counter:stagereversal:featureshape | 0.011 | 0.242 | -0.278 | 0.324 | -0.130 | 0.145 |
| subjTypemonkey:stagereversal:featuredirection | 0.280 | 0.613 | -1.330 | 1.668 | -0.051 | 0.467 |
| subjTypemonkey:stagereversal:featureshape | 0.717 | 0.502 | -0.500 | 1.919 | 0.509 | 0.790 |
| z.cycle_counter:subjTypemonkey:trainingTypeSC2_CDS | -0.492 | 0.449 | -1.609 | 0.612 | -0.768 | -0.314 |
| z.cycle_counter:subjTypemonkey:trainingTypeSC3_DCS | 0.163 | 0.355 | -0.582 | 0.912 | -0.036 | 0.266 |
| z.cycle_counter:stagereversal:trainingTypeSC2_CDS | -0.261 | 0.337 | -0.988 | 0.542 | -0.433 | -0.113 |
| z.cycle_counter:stagereversal:trainingTypeSC3_DCS | 0.103 | 0.268 | -0.450 | 0.668 | -0.007 | 0.290 |
| subjTypemonkey:stagereversal:trainingTypeSC2_CDS | -0.252 | 0.524 | -1.514 | 0.946 | -0.457 | 0.223 |

| | | | | | | |
|---|---|---|---|---|---|---|
| subjTypemonkey:stagereversal:trainingTypeSC3_DCS | 0.162 | 0.442 | -0.950 | 1.192 | -0.089 | 0.378 |
| z.cycle_counter:featuredirection:trainingTypeSC2_CDS | 0.099 | 0.440 | -0.907 | 1.084 | -0.172 | 0.412 |
| z.cycle_counter:featureshape:trainingTypeSC2_CDS | -0.982 | 0.331 | -1.840 | -0.233 | -1.168 | -0.812 |
| z.cycle_counter:featuredirection:trainingTypeSC3_DCS | 0.056 | 0.353 | -0.731 | 0.801 | -0.144 | 0.319 |
| z.cycle_counter:featureshape:trainingTypeSC3_DCS | 0.318 | 0.265 | -0.338 | 0.901 | 0.238 | 0.384 |
| subjTypemonkey:featuredirection:trainingTypeSC2_CDS | -0.636 | 0.757 | -2.142 | 0.801 | -0.854 | -0.381 |
| subjTypemonkey:featureshape:trainingTypeSC2_CDS | 0.581 | 0.835 | -0.491 | 1.674 | 0.360 | 1.283 |
| subjTypemonkey:featuredirection:trainingTypeSC3_DCS | -0.210 | 0.663 | -1.299 | 0.889 | -0.413 | -0.012 |
| subjTypemonkey:featureshape:trainingTypeSC3_DCS | 1.033 | 0.774 | -0.434 | 2.498 | 0.884 | 1.333 |
| stagereversal:featuredirection:trainingTypeSC2_CDS | 0.385 | 0.422 | -0.578 | 1.368 | 0.272 | 0.770 |
| stagereversal:featureshape:trainingTypeSC2_CDS | 0.679 | 0.357 | -0.163 | 1.606 | 0.380 | 0.820 |
| stagereversal:featuredirection:trainingTypeSC3_DCS | 0.500 | 0.338 | -0.323 | 1.306 | 0.344 | 0.643 |
| stagereversal:featureshape:trainingTypeSC3_DCS | 0.683 | 0.291 | -0.036 | 1.432 | 0.546 | 0.776 |
| z.cycle_counter:subjTypemonkey:stagereversal:featuredirection | 0.335 | 0.875 | -2.193 | 2.559 | 0.135 | 0.841 |
| z.cycle_counter:subjTypemonkey:stagereversal:featureshape | -0.002 | 0.581 | -0.011 | 0.008 | -0.163 | 0.387 |
| z.cycle_counter:subjTypemonkey:stagereversal:trainingTypeSC2_CDS | 0.267 | 0.652 | -1.153 | 1.776 | 0.093 | 0.789 |
| z.cycle_counter:subjTypemonkey:stagereversal:trainingTypeSC3_DCS | -0.433 | 0.528 | -1.521 | 0.640 | -0.580 | 0.015 |
| z.cycle_counter:subjTypemonkey:featuredirection:trainingTypeSC2_CDS | -0.801 | 0.898 | -3.129 | 1.399 | -1.098 | -0.501 |
| z.cycle_counter:subjTypemonkey:featureshape:trainingTypeSC2_CDS | 1.092 | 0.707 | -0.648 | 2.823 | 0.921 | 1.972 |
| z.cycle_counter:subjTypemonkey:featuredirection:trainingTypeSC3_DCS | -0.404 | 0.737 | -2.094 | 1.388 | -0.634 | -0.132 |

| | | | | | | |
|---|---|---|---|---|---|---|
| z.cycle_counter:subjTypemonkey:featureshape:trainingTypeSC3_DCS | -0.289 | 0.585 | -1.441 | 0.966 | -0.450 | -0.209 |
| z.cycle_counter:stagereversal:featuredirection:trainingTypeSC2_CDS | -0.964 | 0.575 | -2.283 | 0.394 | -1.283 | -0.488 |
| z.cycle_counter:stagereversal:featureshape:trainingTypeSC2_CDS | 0.706 | 0.416 | -0.188 | 1.562 | 0.630 | 1.007 |
| z.cycle_counter:stagereversal:featuredirection:trainingTypeSC3_DCS | -0.186 | 0.463 | -1.186 | 0.871 | -0.367 | 0.066 |
| z.cycle_counter:stagereversal:featureshape:trainingTypeSC3_DCS | -0.275 | 0.311 | -0.883 | 0.405 | -0.406 | -0.133 |
| subjTypemonkey:stagereversal:featuredirection:trainingTypeSC2_CDS | -0.767 | 0.906 | -2.897 | 1.532 | -1.968 | -0.376 |
| subjTypemonkey:stagereversal:featureshape:trainingTypeSC2_CDS | -0.730 | 0.725 | -2.463 | 0.996 | -2.158 | -0.452 |
| subjTypemonkey:stagereversal:featuredirection:trainingTypeSC3_DCS | -0.615 | 0.757 | -2.510 | 1.335 | -0.795 | -0.320 |
| subjTypemonkey:stagereversal:featureshape:trainingTypeSC3_DCS | -1.336 | 0.622 | -2.847 | 0.272 | -1.622 | -1.087 |
| z.cycle_counter:subjTypemonkey:stagereversal:featuredirection:trainingTypeSC2_CDS | 0.573 | 1.232 | -2.621 | 4.135 | -0.332 | 1.036 |
| z.cycle_counter:subjTypemonkey:stagereversal:featureshape:trainingTypeSC2_CDS | -1.224 | 0.873 | -3.076 | 0.518 | -2.538 | -0.985 |
| z.cycle_counter:subjTypemonkey:stagereversal:featuredirection:trainingTypeSC3_DCS | -0.448 | 1.019 | -3.099 | 2.192 | -0.951 | -0.135 |
| z.cycle_counter:subjTypemonkey:stagereversal:featureshape:trainingTypeSC3_DCS | 0.295 | 0.686 | -0.641 | 1.264 | -0.098 | 0.511 |

**Supplementary Table 1.** Results of the full second-step model (estimated together with their standard errors, 95% confidence limits, and range of estimates when dropping individuals one at a time). (1) Cycle counter was z-transformed, mean and standard deviation of the original cycle counter were 21.7 and 15.3, respectively; subjType was dummy coded with human being the reference level; stage was dummy coded with discrimination being the reference level; feature was dummy coded with color being the reference level; trainingType was dummy coded with SC1_SCD (referred as AUT1 in the text) being the reference level.

Legend for terms:
*z.cycle_counter:* z transformed cycle_counter, which is the counter for discrimination and reversal pairs.
*subjType:* the species (monkey or human)
*stage:* discrimination or reversal
*feature:* shape, color or motion
*trainingType:* the order of which the feature dimensions are introduced (SC1_SCD for shape, color, motion; SC2_CDS, for color, motion, shape; SC3_DCS for motion, color, shape)

0
1

| Animal ID | Number of days in total | Average session duration (minutes) | Training completed on day | Average number of trials | Average duration of breaks | Age | Weight | Training version |
|---|---|---|---|---|---|---|---|---|
| bac | 5 | 71 | 2 | 114 | 249 | 17 | 10 | 2 |
| chi | 5 | 106 | 2 | 154 | 64 | 13 | 8 | 3 |
| cla | 8 | 131 | 3 | 96 | 55 | 7 | 10 | 1 |
| cor | 9 | 113 | 3 | 50 | 124 | 15 | 11 | 1 |
| ear | 9 | 114 | 4 | 57 | 140 | 12 | 12 | 2 |
| elm | 5 | 156 | 1 | 119 | 64 | 11 | 14 | 1 |
| hum | 4 | 141 | 1 | 192 | 37 | 16 | 15 | 3 |
| ken | 5 | 107 | 4 | 66 | 109 | 8 | 7 | 3 |
| lin | 5 | 106 | 3 | 60 | 163 | 15 | 10 | 2 |
| pin | 4 | 95 | 1 | 96 | 132 | 12 | 11 | 2 |
| vin | 5 | 191 | 4 | 37 | 131 | 9 | 6 | 3 |
| average | 6 | 121 | 2.5 | 95 | 115 | 12 | | |

**Supplementary Table 2.** The table summarizes, for each animal, some descriptive information on their task performance and demographics such as age and weight. As the task was composed of two parts, training and testing, the column 'training completed on day' refers to the day that the first part of the experiment was completed and the animals went further with the second, testing phase.
